# Genome-Wide Identification of the GELP Family in *Juglans mandshurica* Reveals Their Potential Roles in Seed Development and Stress Responses

**DOI:** 10.3390/ijms27156557

**Published:** 2026-07-23

**Authors:** Meng Dang, Rui Wang, Zhenlin Shen, Fan Wu, Changcong Yan, Qianyu Wang, Huijuan Zhou

**Affiliations:** 1 Eco-Environmental Conservation Research Centre of Xin’an River Basin, College of Life and Environmental Sciences, Huangshan University, Huangshan City 245021, China; dangmeng13@163.com; 2 Key Laboratory of Resource Biology and Biotechnology in Western China, Ministry of Education, College of Life Sciences, Northwest University, Xi’an 710069, China; shqyxlb2020@163.com (R.W.); szl0602@163.com (Z.S.); 202421758@stumail.nwu.edu.cn (F.W.); 2023113090@stumail.nwu.edu.cn (C.Y.); monica91995636@163.com (Q.W.); 3 Xi’an Botanical Garden of Shaanxi Province, Institute of Botany of Shaanxi Province, Shaanxi Academy of Science, Xi’an 710061, China

**Keywords:** GELP family, genome-wide analysis, expression profiling, seed development, stress responses, *Juglans mandshurica*

## Abstract

GDSL esterase/lipases (GELPs) are important regulators of plant growth and development, lipid metabolism, and stress responses. However, their genomic characteristics and expression patterns have not been systematically characterized for *Juglans mandshurica*, a woody oil crop species of significant ecological and economic value. Here, we identified 61 *JmGELP* genes in *J. mandshurica* through genome-wide analysis. Phylogenetic analysis classified them into seven major clades, and variations in gene structure and conserved motifs suggested potential functional divergence. Promoter cis-acting element analysis revealed widespread enrichment of motifs responsive to light, phytohormones, and abiotic stresses. Transcriptomic sequencing and qRT-PCR validation revealed distinct tissue-specific and seed development stage-specific expression patterns of *JmGELP* members, as well as their differential responses to various stress and hormone treatments. Gene Ontology (GO) annotation and protein–protein interaction (PPI) network analyses further supported their involvement in lipid metabolism. In silico analyses of transcription factor binding sites, miRNA targets, and molecular docking predicted that *JmGELP-3*, *-38*, and *-41* have distinct transcriptional and post-transcriptional regulatory networks and potentially divergent substrate preferences. This study provides the first comprehensive characterization of the GELP family in *J. mandshurica*, identifying candidate genes that may inform future germplasm improvement and stress-resistance breeding in *Juglans* species.

## 1. Introduction

The GDSL-type esterase/lipase (GELP) gene family, defined by the conserved Gly-Asp-Ser-Leu (GDSL) motif, is widely distributed across diverse organisms [1]. Family members share five conserved blocks (I–V) within their primary structure. Four highly conserved amino acid residues at the catalytic site are Serine (Ser) in block I, Glycine (Gly) in block II, Asparagine (Asn) in block III and Histidine (His) in block V [2]. The characteristic GDSL motif resides in block I, and together Ser, His, and Asp form a catalytic triad that is essential for enzymatic activity [3].

The GELP family is ubiquitous across eukaryotes and prokaryotes and represents a large, diverse multigene family in plants [4,5]. Advances in genome sequencing and bioinformatics have enabled genome-wide identification of this family in numerous plant species [6,7,8,9]. For instance, 114, 105, 87, 80, and 75 GELP family members have been identified in *Oryza sativa* [10], *Arabidopsis thaliana* [11], *Carya illinoensis* [12], *Solanum lycopersicum* [13], and *Cucumis sativus* [9], respectively. These members play crucial roles in plant growth and development [7,14,15], lipid metabolism [16,17], and stress responses [18,19]. For example, *Arabidopsis SFAR4* (*AtGELP1*) regulates seed germination by hydrolyzing abscisic acid-glucose ester to modulate hormone homeostasis [14], whereas *CaGLIP1* in pepper coordinates disease resistance and drought tolerance [18]. Many GELPs are also integrated into hormone signaling pathways, especially those involving jasmonic acid (JA) and salicylic acid (SA), and thereby mediate both biotic and abiotic stress responses [13]. However, no systematic investigation of the GELP family has been conducted in *Juglans mandshurica*, leaving its composition, expression profiles, and stress-related functions largely unexplored.

*Juglans mandshurica* is a highly valuable hardwood species prized for its durable, corrosion-resistant timber [20,21]. This diploid species (2n = 32) [22] serves as a premium raw material for high-end furniture, military equipment, and shipbuilding. Moreover, various tissues of *J. mandshurica* produce bioactive compounds, including juglone, which has shown significant anti-tumor potential [20]. The species also exhibits strong adaptability to cold, drought, and salt stresses, making it an ideal rootstock resource for economically important species such as *Juglans regia* [23,24]. The recent availability of a high-quality genome sequence [25] now enables genome-wide investigation of gene families associated with stress resistance and secondary metabolism.

Here, we report the first genome-wide identification and comprehensive bioinformatic analysis of the GELP gene family in *J. mandshurica*. We systematically characterized the family members with respect to phylogeny, chromosomal distribution, gene duplication, conserved motifs, and cis-regulatory elements. Using transcriptome data from diverse tissues and developing seeds, we analyzed the expression profiles of *JmGELP* genes to identify candidates that may be involved in organ development, seed oil metabolism, and stress responses. Collectively, our findings enhance the current understanding of the genetic basis underlying lipid metabolism and stress adaptation in *J. mandshurica*. Importantly, they also provide candidate targets for molecular breeding to modulate secondary metabolism and improve stress tolerance.

## 2. Results

### 2.1. Identification and Physicochemical Characterization of the GELP Gene Family in Juglans mandshurica

**Figure 1:**
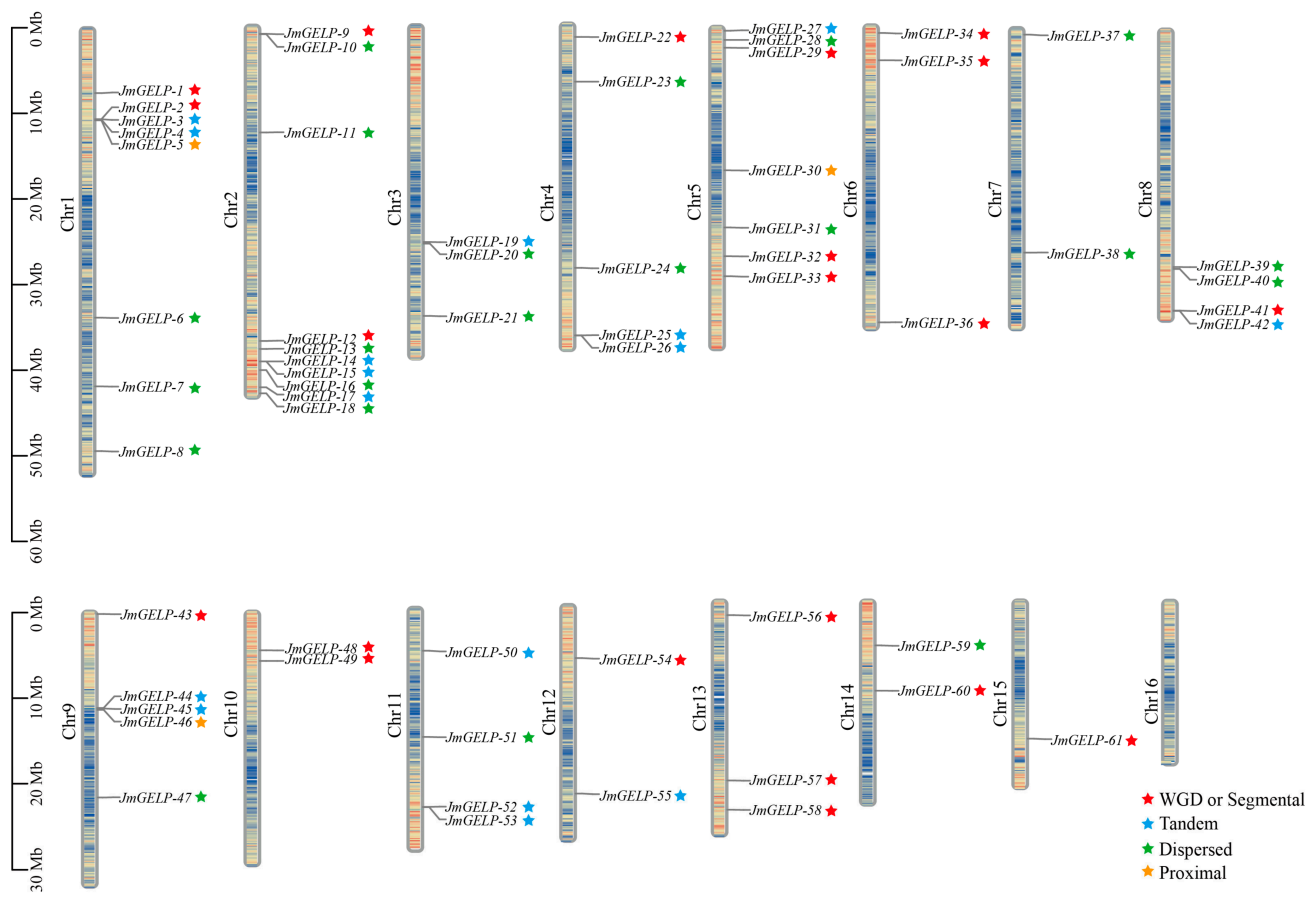
Chromosomal distribution and duplication patterns of *JmGELP* genes in *J. mandshurica*. Chromosomes are represented by vertical bars with chromosome numbers labeled on the left. Gene positions are indicated by colored stars: red, whole-genome (WGD) or segmental duplication; blue, tandem duplication; green, dispersed duplication; orange, proximal duplication.

By aligning the GELP protein sequences of *Arabidopsis thaliana* against the *J. mandshurica* genome, we identified 61 *JmGELP* family members based on their conserved domain characteristics, which were systematically named *JmGELP-1* to *JmGELP-61*. Their physicochemical properties, including amino acid length, molecular weight, isoelectric point, instability index, aliphatic index, grand average of hydropathicity, and subcellular localization, are summarized in Table 1. Detailed information on gene names, gene and protein accession IDs, and chromosomal locations for each member is provided in Table S1. These genes were unevenly distributed across 15 chromosomes. Chromosome 2 contained the most members (9 genes), while chromosome 15 contained the fewest (1 gene). Chromosomes 1, 2, and 5 collectively harbored 25 *JmGELP* genes, accounting for 40.98% of the total family (Figure 1; Table S1). The JmGELP proteins showed diversity in length (338–551 amino acids) and molecular weight (36.6–58.7 kDa). Most members (55 proteins) were clustered within the range of 350–430 amino acids. Isoelectric point (pI) values ranged from acidic (4.23) to basic (9.37), indicating considerable charge heterogeneity among the family members. Proteins encoded by genes located on the same chromosome also displayed a broad pI range. Instability index predictions showed that all members except JmGELP-26 (48.27), JmGELP-29 (40.9), JmGELP-51 (67.53), and JmGELP-59 (45.85) are stable proteins (with an instability index < 40 defined as the stability threshold), suggesting that most JmGELPs are likely to maintain structural integrity under physiological conditions. Approximately 62.3% of the proteins (38 members) had negative grand average of hydropathicity (GRAVY) values, reflecting a predominantly hydrophilic nature. The median aliphatic index was 85.5, implying moderate thermostability. Subcellular localization was predicted for each JmGELP protein. The vast majority of JmGELP members (57 proteins, 93.44%) were predicted to be localized to the extracellular region. JmGELP-20 and JmGELP-50 were predicted to be targeted to mitochondria, JmGELP-27 to chloroplasts, and JmGELP-59 to vacuoles.

### 2.2. Comparative Phylogenetic Analysis of the JmGELP and AtGELP Families

**Figure 2:**
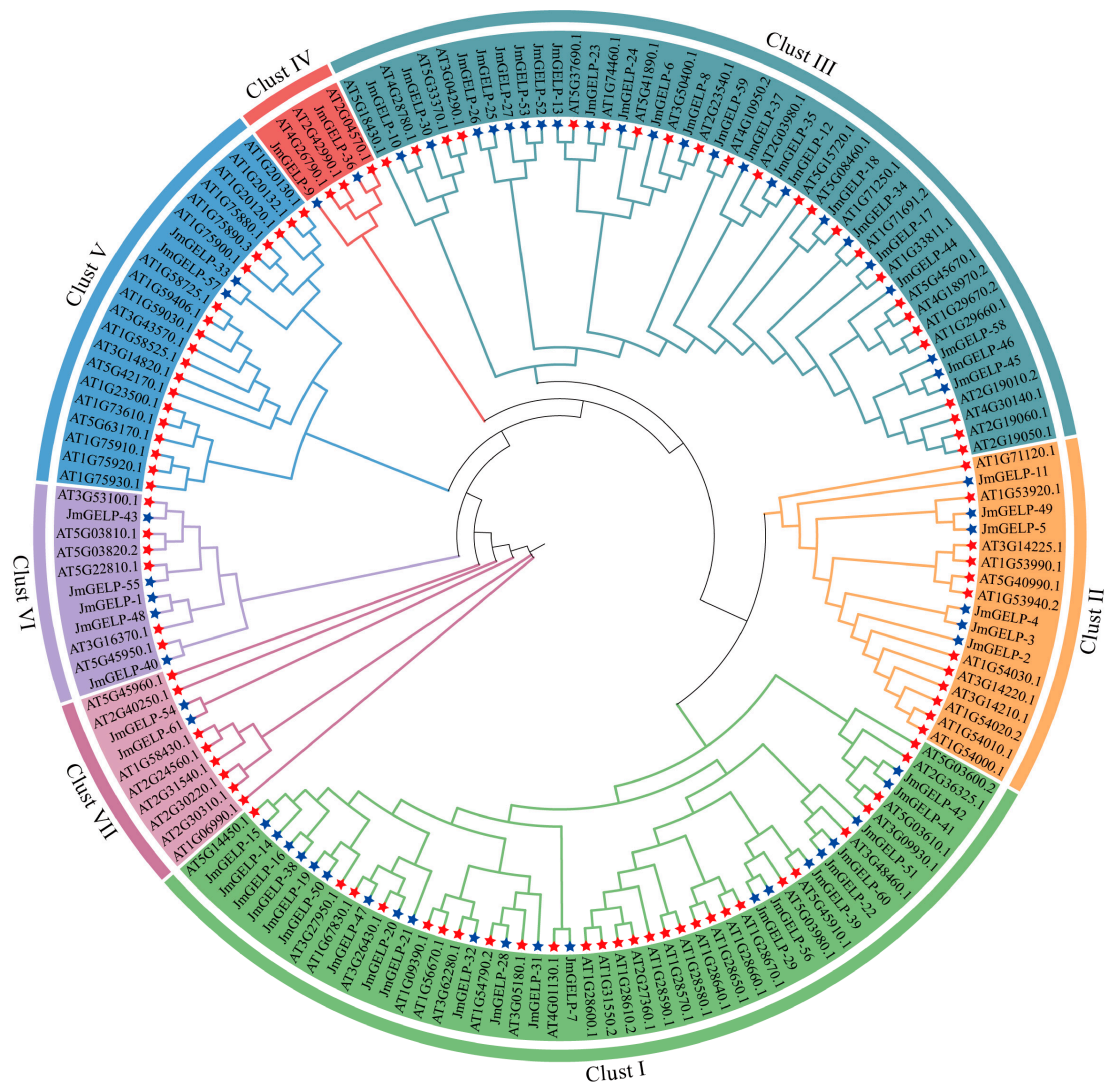
Phylogenetic analysis of GELP proteins from *J. mandshurica* (JmGELP) and *A. thaliana* (AtGELP). JmGELP proteins are indicated by blue stars, and AtGELP proteins by red stars. The seven major clades (I–VII) are color-coded. The maximum likelihood tree was constructed using the JTT + R10 substitution model with 1000 ultrafast bootstrap replicates.

A Maximum Likelihood phylogenetic tree was constructed using 61 JmGELP proteins together with their 100 homologs from *A. thaliana*. Based on the JTT + R10 substitution model and 1000 ultrafast bootstrap replicates, the ML phylogeny classified all GELP proteins into seven monophyletic subclades (Clust I–VII), each receiving moderate to strong statistical support (bootstrap ≥ 70%) (Figure 2). Clust I was the largest subclade, encompassing 21 JmGELPs (34.4% of the total JmGELPs) and 28 AtGELPs; Clust II contained 6 JmGELPs (9.8%) and 12 AtGELPs; Clust III comprised 23 JmGELPs (37.7%) and 24 AtGELPs; Clust IV included 2 JmGELPs and 3 AtGELPs; Clust V contained 2 JmGELPs and 19 AtGELPs; Clust VI comprised 5 JmGELPs and 6 AtGELPs; and Clust VII included 2 JmGELPs and 8 AtGELPs.

### 2.3. Structural Architecture of JmGELP Proteins

**Figure 3:**
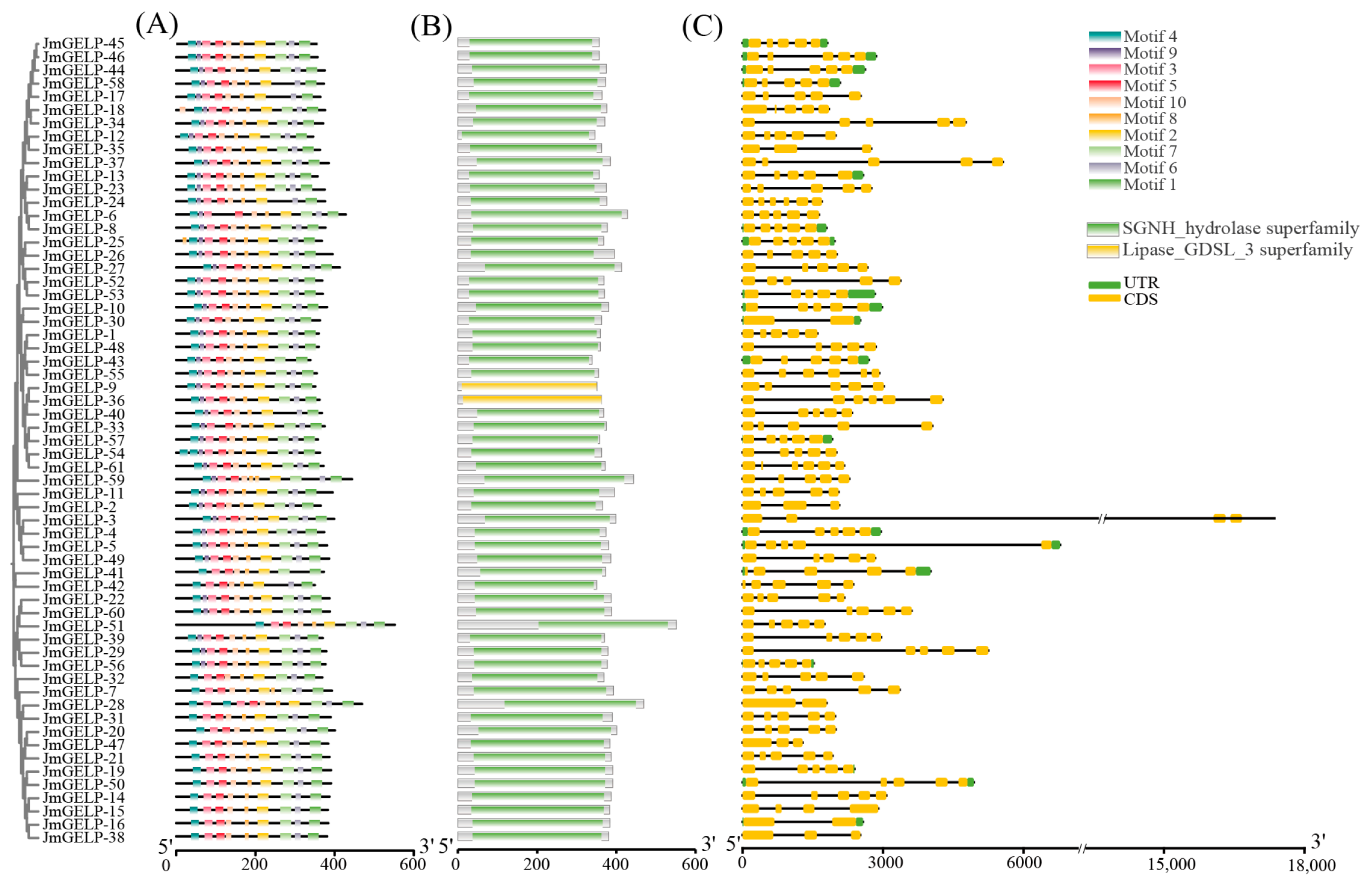
Conserved motifs and gene structures of JmGELP proteins. (**A**) Distribution of conserved motifs across JmGELP proteins (motif numbers indicated). (**B**) Schematic representation of protein domains. (**C**) Gene structures: green boxes, coding sequences (CDS); yellow boxes, untranslated regions (UTR); black lines, introns. Scale bars indicate sequence lengths.

The structural features of the JmGELP gene family, including conserved motifs, protein domains, and gene structure, were characterized. MEME analysis identified ten conserved motifs (Motif 1–10) (Figure 3A). Among them, Motifs 4, 3, 8, and 1, corresponding to the core catalytic blocks (I, II, III, V) of GELP proteins, were present in all 61 members. In contrast, Motif 9 was absent in most members of Clade I (Figure 3A). Domain annotation showed that all 61 JmGELP members belong to the GDSL/SGNH hydrolase superfamily (Figure 3B). Most members (except JmGELP-9 and JmGELP-36) were annotated with the SGNH_hydrolase superfamily domain. JmGELP-9 and JmGELP-36 were annotated with the Lipase_GDSL_3 superfamily domain, which is an alternative nomenclature in the Pfam database. All identified motifs were located within these domain regions (Figure 3B). Gene structure analysis showed that most *JmGELP* members share a conserved arrangement of five exons and four introns (Figure 3C). Despite this conserved exon number, variation in exon length and positioning was observed.

### 2.4. Gene Duplication and Synteny Analysis of JmGELP Genes

**Figure 4:**
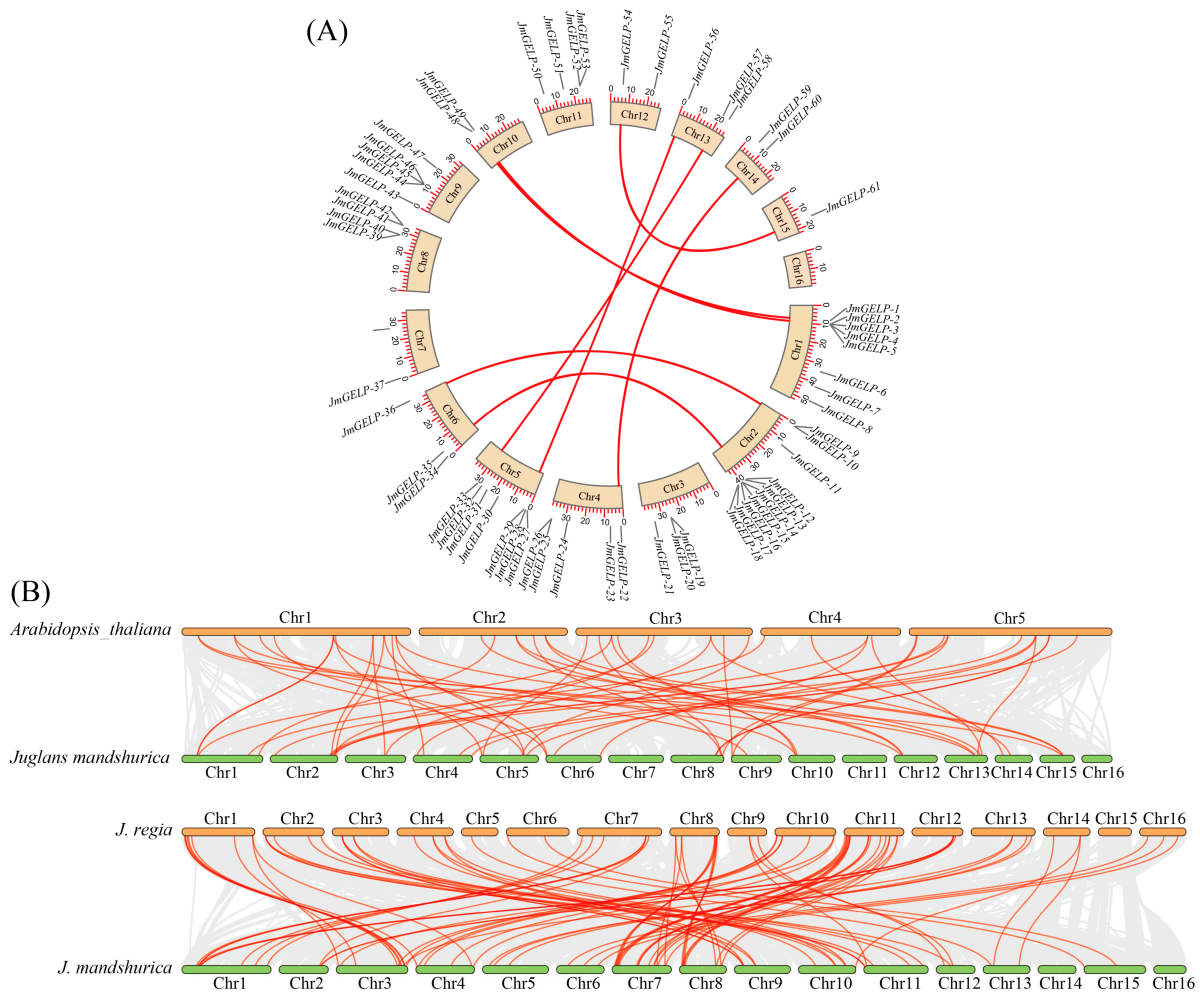
(**A**) Collinearity analysis of GELP genes in *J. mandshurica*. Red lines highlight segmentally duplicated GELP gene pairs. (**B**) Synteny analysis of GELP genes between *J. mandshurica* and *A. thaliana*, and between *J. mandshurica* and *regia*. Gray lines represent systemic regions across the two genomes; red lines highlight syntenic *GELP* gene pairs.

Analysis of genomic duplication events revealed the distribution of the 61 *JmGELP* genes among different duplication types. Whole-genome/segmental duplication (WGD/Segmental) and dispersed duplication (DSD) each accounted for 34.4% (21 genes) of the family, while tandem duplication (TD) contributed 26.2% (16 genes) and proximal duplication (PD) accounted for 5.0% (3 genes) (Figure 1 and Table S2). Eight pairs of duplicated *JmGELP* genes were identified (*JmGELP-1*/*48*, *JmGELP-2*/*49*, *JmGELP-9*/*36*, *JmGELP-12*/*35*, *JmGELP-22*/*60*, *JmGELP-29*/*56*, *JmGELP-33*/*57*, and *JmGELP-54*/*61*) (Figure 4A and Table S3). The non-synonymous (Ka) and synonymous (Ks) substitution rates were calculated for these eight pairs, with values ranging from 0.20 to 0.37 (Table S3). All ratios were significantly less than 1. For 50 syntenic GELP gene pairs between *J. mandshurica* and *A. thaliana*, the mean Ka/Ks ratio was 0.16 (Figure 4B and Table S4). Furthermore, synteny analysis between *J. mandshurica* and *J. regia* (common walnut) identified 69 syntenic GELP gene pairs (Table S5). The Ka/Ks values for these 69 pairs ranged from 0.054 to 1.147, with a median of 0.285; the vast majority (68 out of 69) were less than 1, indicating predominantly purifying selection (Figure 4B and Table S5). The much higher number of syntenic pairs compared with *A. thaliana* reflects the closer phylogenetic relationship between the two *Juglans* species.

### 2.5. Analysis of Cis-Acting Elements in JmGELP Gene Promoters

**Figure 5:**
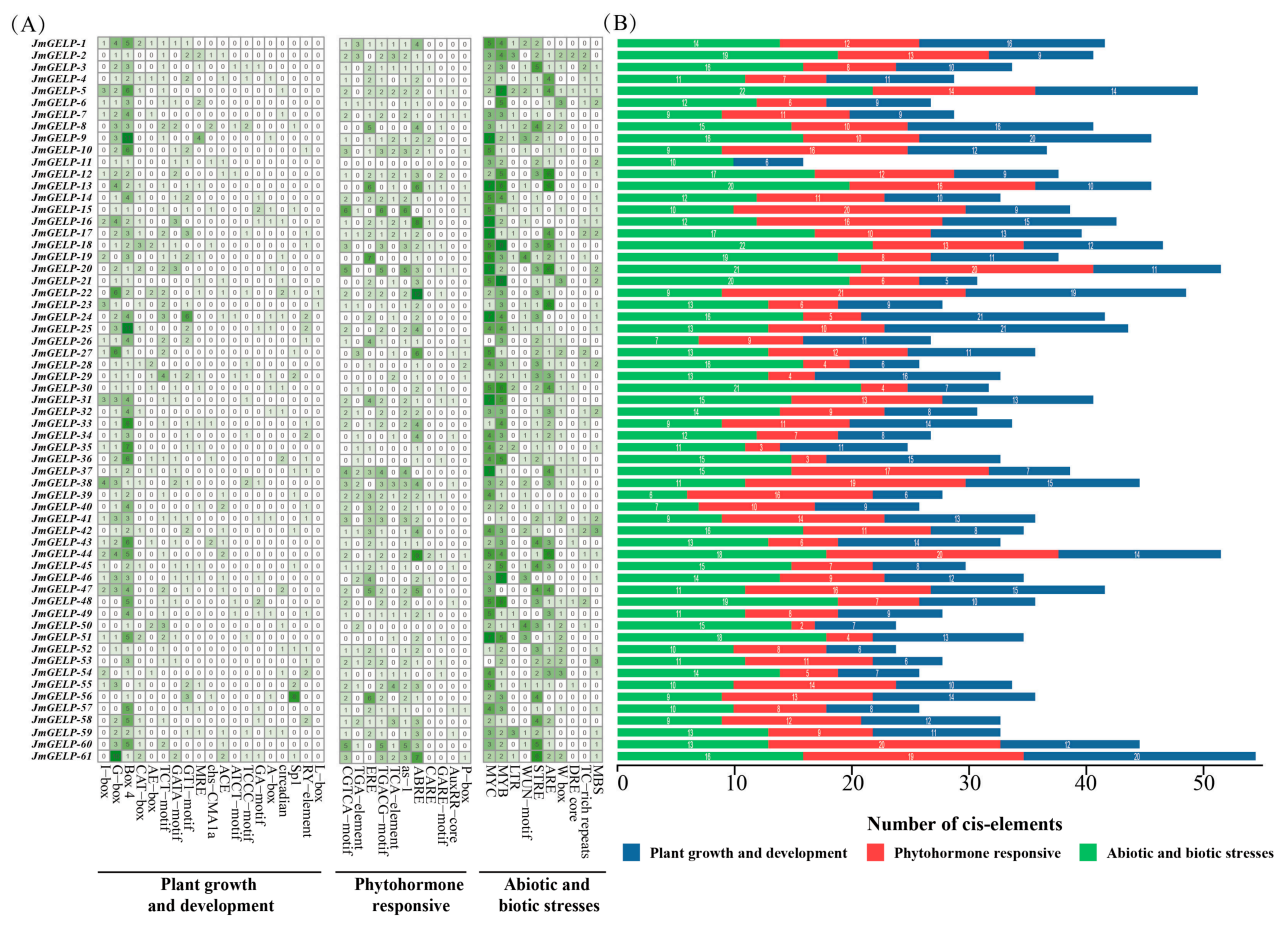
Cis-acting elements in the promoter regions of *JmGELP* genes. (**A**) Numbers of cis-acting elements in each gene promoter. (**B**) Distribution of elements by functional category. The x-axis indicates the number of elements per type within the 2.0 kb promoter region. Bars are stacked and color-coded by function: blue, plant growth and development; red, phytohormone responsiveness (e.g., ABRE, CGTCA-motif); green, abiotic and biotic stress responses (e.g., DRE, LTR, W-box).

The 2.0 kb promoter regions of the 61 *JmGELP* genes were analyzed, revealing a diverse array of cis-acting elements that were classified into three functional categories: plant growth and development, phytohormone responsiveness, and abiotic/biotic stress responses (Figure 5). The total number of elements per gene varied considerably (Figure 5B). Light-responsive elements (G-box and Box 4) were detected in most promoters; notably, Box 4 was particularly abundant in *JmGELP-9* and *JmGELP-25*. Other developmental elements (CAT-box, RY-element, and circadian elements) were present in a subset of promoters. For instance, the RY-element was present in *JmGELP-24*, *JmGELP-25*, *JmGELP-34*, *JmGELP-54*, and *JmGELP-58*. Hormone-responsive elements were also enriched in the promoters. Jasmonic acid-responsive motifs (CGTCA-motif, TGACG-motif) were widespread, with particularly high density in *JmGELP-15* and *JmGELP-20*. The abscisic acid-responsive ABRE element was abundant, especially in *JmGELP-1*, *JmGELP-16*, *JmGELP-22*, and *JmGELP-44*. Stress-responsive elements, including MYB and MYC binding sites and the anaerobic response element (ARE), were also prevalent. MYB sites were particularly abundant in *JmGELP-5*, *JmGELP-21*, and *JmGELP-46*, while ARE was enriched in *JmGELP-4*, *JmGELP-5*, *JmGELP-12*, and *JmGELP-20*.

### 2.6. Expression Profiling of JmGELP Genes Across Tissues and During Seed Development

RNA-seq data from three major tissues (bark, leaf, and fruit pericarp) and during four stages of seed development (S1–S4) were analyzed (Table S6). Using stringent differential expression criteria (|log_2_FC| ≥ 1, FDR ≤ 0.05), 26 *JmGELP* genes were differentially expressed across the three tissues (Table S7). These tissue expression data were used for initial candidate gene screening. Separately, 43 *JmGELP* genes showed significant expression changes during seed development (Table S8).

**Figure 6:**
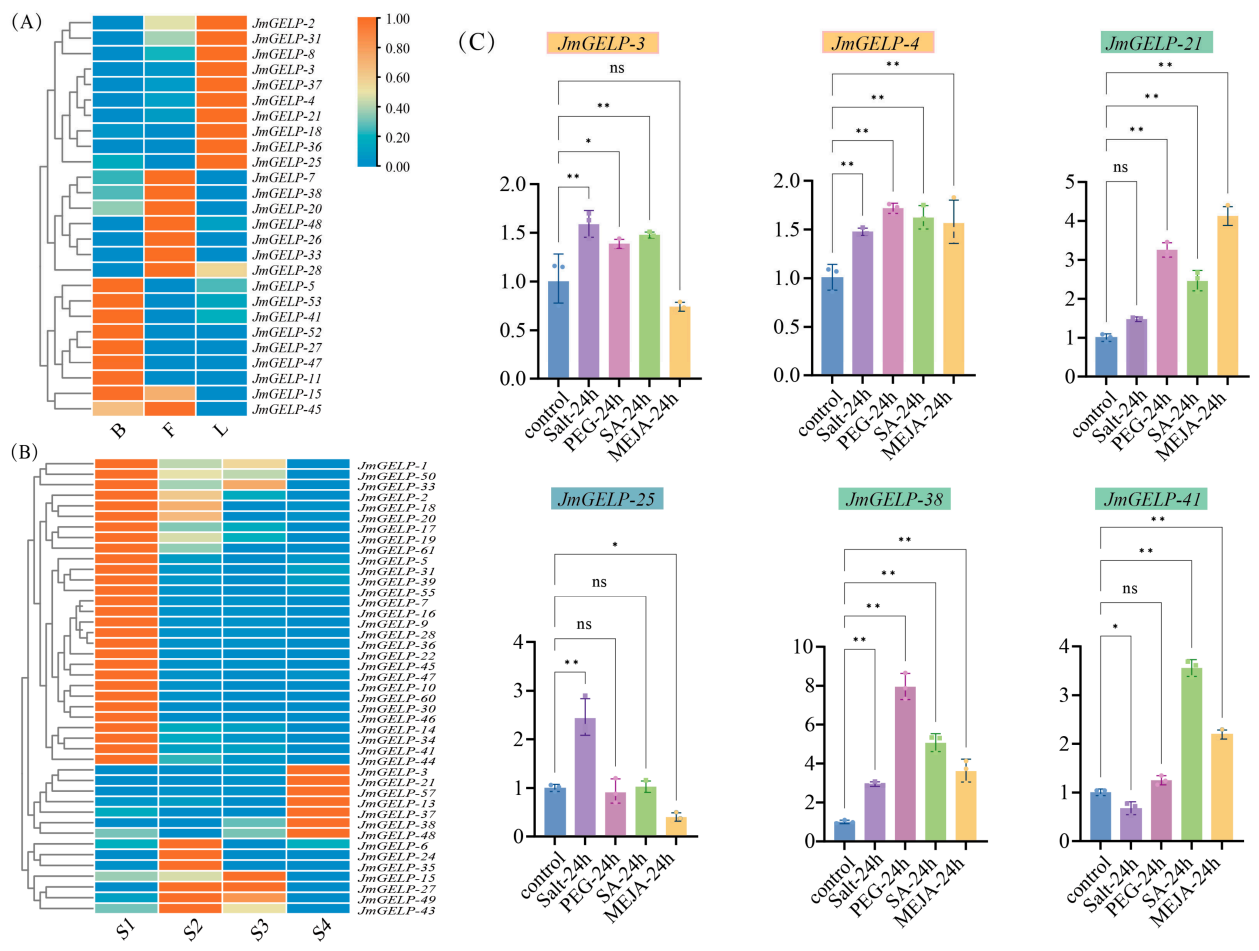
Expression patterns of *JmGELP* genes under different conditions. (**A**) Transcript levels in bark (B), green fruit pericarp (F), and leaf (L) tissues. (**B**) Expression profiles during four seed developmental stages (S1–S4). Both heatmaps display Z-score normalized TPM values of *JmGELP* genes that were differentially expressed (|log_2_FC| ≥ 1, FDR ≤ 0.05) in at least one pairwise comparison (across tissues for (**A**); across stages for (**B**)). (**C**) qRT-PCR validation of six *JmGELP* genes after 24 h of control, NaCl (200 mM), PEG (20% *w*/*v*), SA (100 μM), and MeJA (100 μM) treatments. Relative expression levels are shown as mean ± SD (n = 3 biological replicates). Statistical significance between each treatment group and the control group was determined by unpaired two-tailed Student’s *t*-test. * *p* < 0.05; ** *p* < 0.01. Background colors of gene names indicate phylogenetic clades: green, Clade I; orange, Clade II; blue, Clade III.

*JmGELP* genes displayed diverse different expression profiles in the three tissues (Figure 6A and Table S7) with some genes showing tissue-preferential expression. *JmGELP-52* and *JmGELP-53* were highly expressed in bark, with Transcripts Per Million (TPM) values of 191.71 and 287.74, respectively. *JmGELP-3* and *JmGELP-21* were highly expressed in green leaves, with TPM values of 112.53 and 137.24. By contrast, other members, such as *JmGELP-38* and *JmGELP-41*, were expressed at relatively high levels across all three tissues.

Transcriptome analysis across four stages of seed development (S1 to S4) revealed distinct temporal expression patterns of *JmGELP* genes (Figure 6B and Table S8). Several expression trends were observed among the differentially expressed members. A group of genes (*JmGELP-16*, *JmGELP-22*, *JmGELP-30*, and *JmGELP-34*) peaked during the initial stages (S1/S2) and then declined. Another group (*JmGELP-13*, *JmGELP-38*, and *JmGELP-48*) was upregulated in the later seed stages (S3/S4). *JmGELP-15* remained consistently high throughout all stages, whereas *JmGELP-33* maintained high expression from S1 to S3 before declining in S4. Most *JmGELP* genes were downregulated in S4.

### 2.7. Differential Response of JmGELP Genes to Abiotic Stress and Hormone Treatments

Based on transcriptome data, six highly expressed genes in leaves (*JmGELP-3*, *-4*, *-21*, *-25*, *-38*, and *-41*) were selected for qRT-PCR validation (Table S9). These leaf-dominant members exhibited differential expression patterns following 24 h treatments with 200 mM NaCl, 20% (*w*/*v*) PEG-6000, 100 μM SA, and 100 μM MeJA (Figure 6C and Table S10). *JmGELP-38* showed the strongest induction, with expression levels increasing by approximately 8.0-fold (PEG), 5.1-fold (SA), and 3.6-fold (MeJA) relative to controls. *JmGELP-21* was also significantly upregulated by MeJA (4.1-fold) and PEG (3.3-fold). *JmGELP-3* and *JmGELP-4* were moderately upregulated by NaCl, PEG, and SA (approximately 1.5-fold). *JmGELP-4* showed a consistently moderate response to MeJA (1.6-fold), whereas *JmGELP-3* was not induced by MeJA (0.7-fold). *JmGELP-25* was upregulated by NaCl (2.5-fold) and downregulated by MeJA (0.4-fold). *JmGELP-41* was upregulated by SA (3.6-fold) and MeJA (2.2-fold), but downregulated by NaCl (0.7-fold).

### 2.8. Protein Interaction Network and Functional Enrichment Analysis of Arabidopsis orthologs of the JmGELP Family

**Figure 7:**
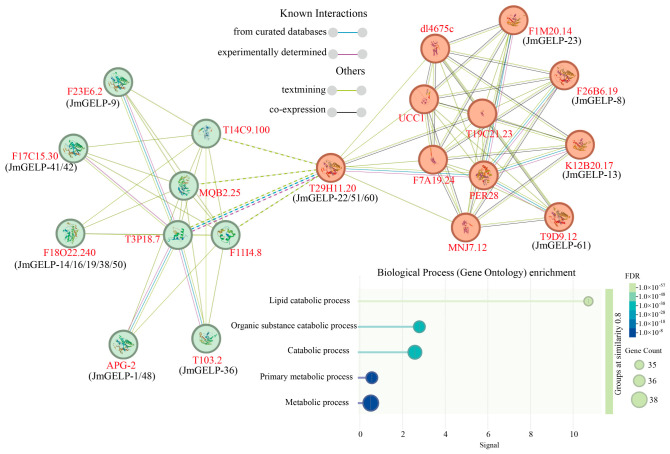
Protein–protein interaction network and functional enrichment of JmGELP proteins based on *A. thaliana* orthologs. High-confidence interaction network (STRING combined score ≥ 0.700). Edges represent interactions supported by experimental, database, textmining and co-expression evidence. The bar chart displays the top enriched GO terms, with the lipid catabolic process highlighted. The false discovery rate (FDR) is indicated.

We constructed a high-confidence protein–protein interaction network using *A. thaliana* orthologs of JmGELP proteins in the STRING database (combined score ≥ 0.700) (Figure 7). Out of 61 JmGELP proteins, 38 (62.3%) were mapped to high-confidence *A. thaliana* orthologs (E-value ≤ 1 × 10^−5^, identity ≥30%) and subjected to subsequent analysis. Among these, eight mapped JmGELP proteins formed the core interaction nodes of the network. Their interactions were supported by multiple types of evidence (curated databases, experimental verification, text mining, and co-expression). These core proteins are: JmGELP-1/48 (*Arabidopsis* ortholog APG-2, AT5G45680), JmGELP-8 (F26B6.19), JmGELP-9 (F23E6.2), JmGELP-13 (K12B20.17), JmGELP-22/51/60 (T29H11.20), JmGELP-23 (F1M20.14), JmGELP-36 (T1O3.2), and JmGELP-61 (T9D9.12). The remaining members were assigned potential interaction partners through homology-based prediction. These interacting partners included multiple functionally annotated proteins. Representative partners include lipid transfer proteins, such as the LTP family members F11I4.8 (AT1G48750) and T3P18.7 (AT1G62510). Another set of proteins related to oxidative polymerization was also identified, including the class III peroxidase PER28 (AT3G03670), the cytochrome b561 proteins dl4675c (AT4G17280) and MNJ7.12 (AT5G47530), and the uclacyanin UCC1 (AT2G32300).

Gene Ontology enrichment analysis of the network returned highly significant results (Figure 7 and Table S11). The network was most strongly enriched for the lipid catabolic process (GO:0016042; FDR = 1.01 × 10^−57^), with 92.1% (35/38) of the genes annotated to this function. Enrichment was also observed for the broader terms organic substance catabolic process (GO:1901575) and catabolic process (GO:0009056).

### 2.9. Prediction of TFs and miRNAs Targeting of JmGELP Members

**Figure 8:**
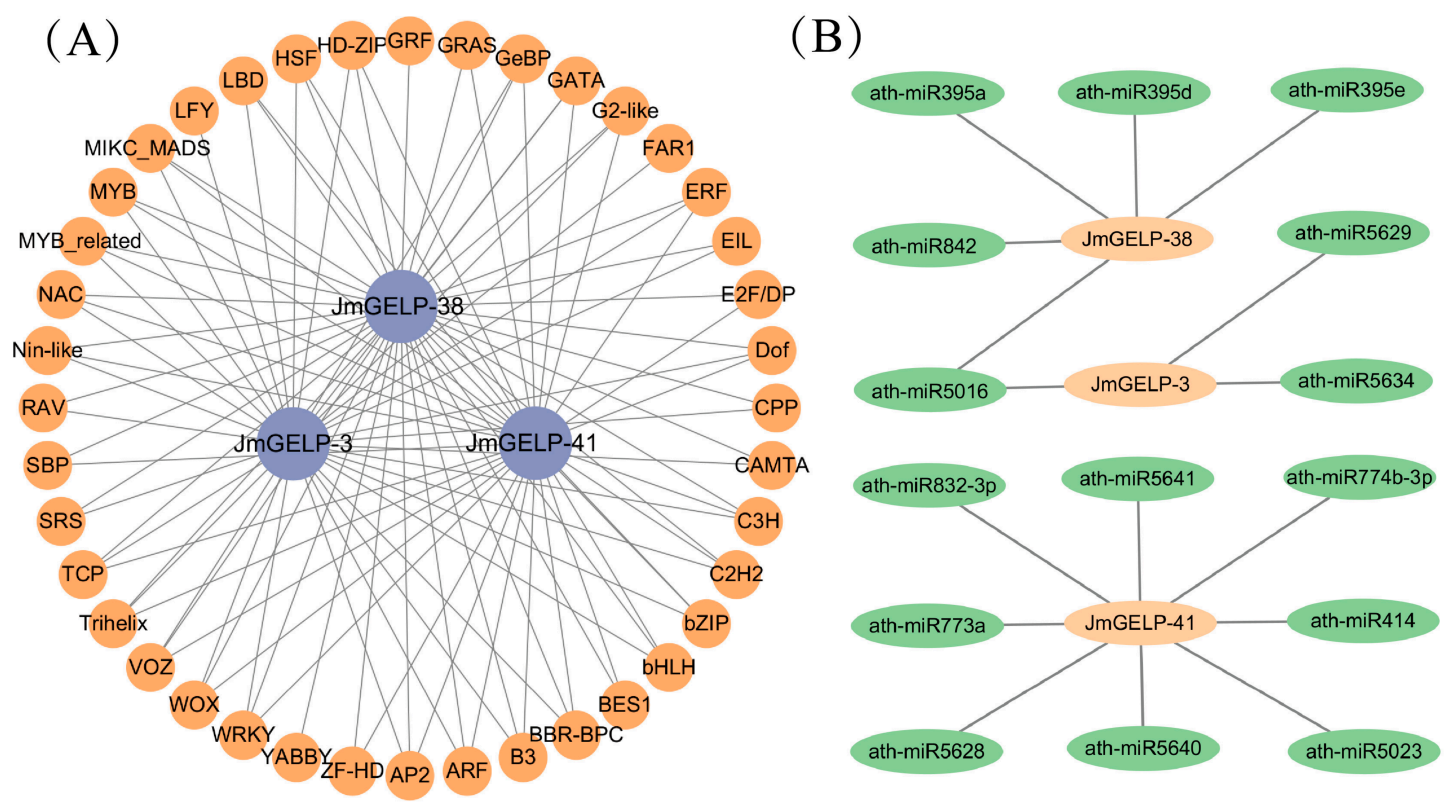
Predicted regulatory networks for three candidate *JmGELP* genes. (**A**) Transcription factor (TF) binding site prediction. Orange circles represent known TFs reported in the literature; purple circles denote the candidate genes *JmGELP-38*, *JmGELP-3*, and *JmGELP-41*. Edges indicate predicted TF-DNA interactions; line thickness does not represent statistical significance. (**B**) Small RNA prediction. Green ellipses represent putative small RNAs predicted to target the mRNA transcripts of the candidate genes. Edges illustrate predicted miRNA-mRNA interactions.

We analyzed the promoter regions of the three candidate genes for potential transcription factor (TF) binding sites. A total of 41 TF families were predicted to bind to these promoter regions, with binding profiles differing among the three genes (Figure 8A and Table S12). Specifically, the promoter region of *JmGELP-38* showed enrichment for binding sites of multiple stress-responsive TFs (e.g., ERF, WRKY, NAC, MYB) and TFs involved in later developmental stages (e.g., MIKC_MADS, WOX). By contrast, the promoter of *JmGELP-41* contained regulatory elements associated with early development (e.g., BBR-BPC, ARF, LBD) and defense responses. *JmGELP-3* had the fewer binding sites than the other two genes, with a binding site composition primarily consisting of elements commonly found in leaf tissues and showing fewer binding signals from stress-responsive TFs.

We also predicted miRNAs that may target the transcripts of the candidate genes (Figure 8B and Table S13). The *JmGELP-38* transcript was predicted to be targeted by multiple miR395 family members (a, d, e) and ath-miR842. *JmGELP-41* was predicted to be targeted by the highest number of small RNAs, including ath-miR414, ath-miR774b-3p, and ath-miR832-3p. *JmGELP-3* had the fewest predicted miRNA target sites.

### 2.10. Molecular Docking Analysis of JmGELP Candidate Genes

**Figure 9:**
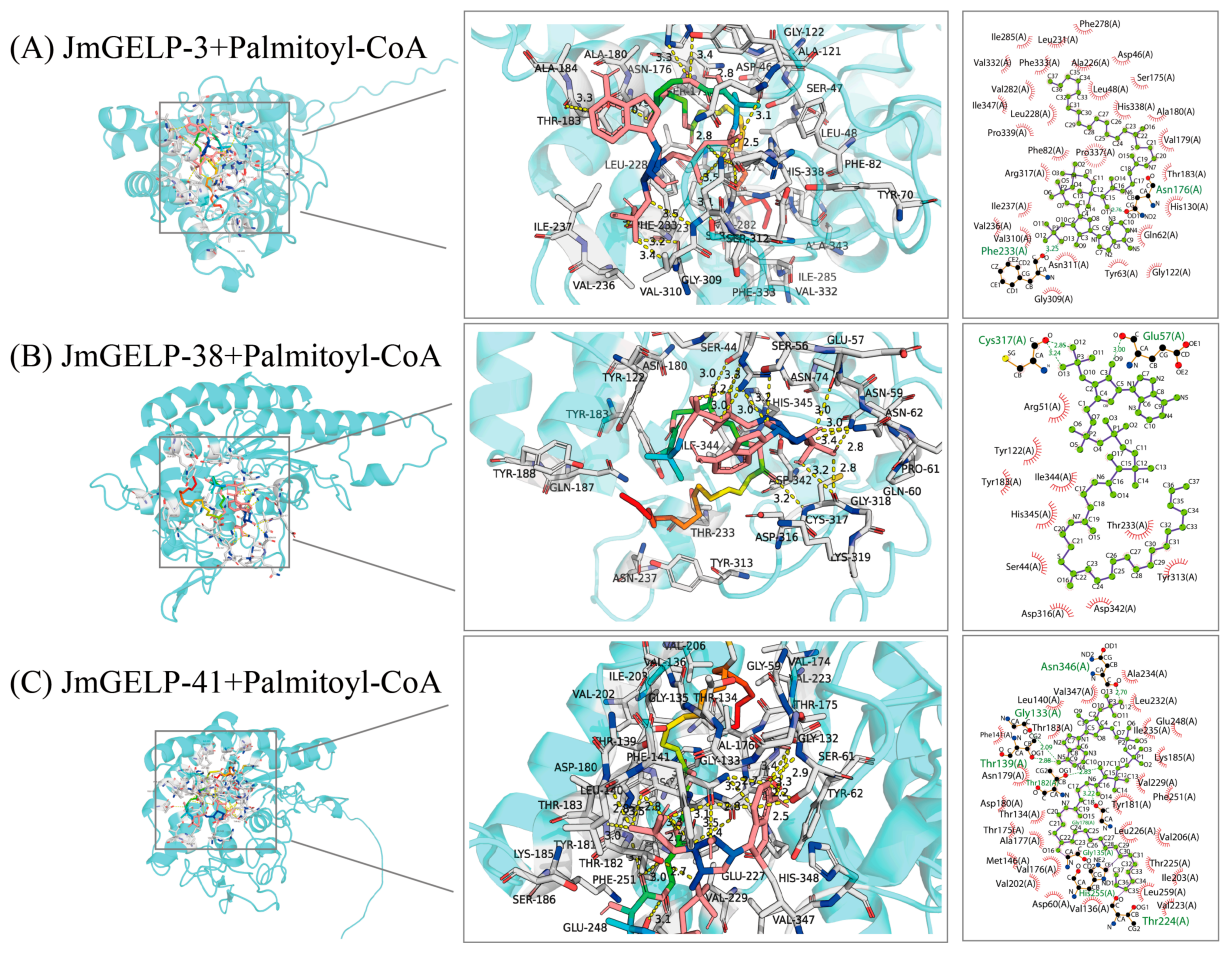
In silico molecular docking of JmGELP-3 (**A**), JmGELP-38 (**B**), and JmGELP-41 (**C**) proteins with palmitoyl-CoA. Left panel shows the overall predicted 3D docking complex (protein ribbon, ligand and catalytic triad as sticks). Middle panel details the interaction interface, including key residues (Ser, Asp, His), binding sites, and the intermolecular distances (Å) between residues and ligand atoms. Right panel shows the 2D ligand structure and interaction types (e.g., hydrogen bonds, hydrophobic contacts).

**Figure 10:**
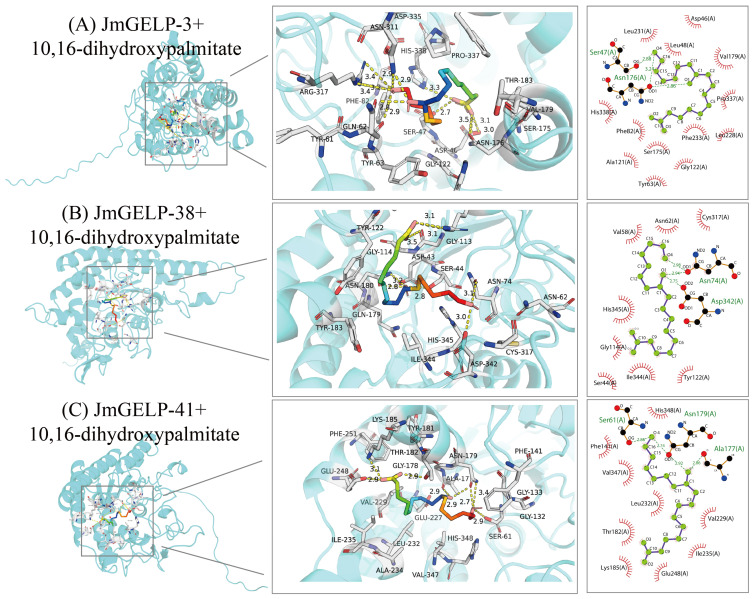
In silico molecular docking of JmGELP-3 (**A**), JmGELP-38 (**B**), and JmGELP-41 (**C**) proteins with 10,16-dihydroxypalmitate. Panel layout and annotations are identical to those in Figure 9.

To explore the potential functional divergence among the key candidate genes *JmGELP-3*, *JmGELP-38*, and *JmGELP-41* in lipid metabolism, we performed molecular docking of their encoded proteins (Figure 9 and Figure 10). All three proteins contain the conserved Ser-Asp-His catalytic triad characteristic of the GELP family. We selected two lipid-related metabolites (palmitoyl-CoA and 10,16-dihydroxypalmitate) that have been implicated in cuticle formation in other species. The docking results showed differences in binding free energy (ΔG) for the three proteins with the two substrates (Table S14). JmGELP-38 was predicted to bind palmitoyl-CoA with a ΔG of −5.94 kcal/mol, and to bind 10,16-dihydroxypalmitate with a ΔG of −4.60 kcal/mol. JmGELP-41 was predicted to bind 10,16-dihydroxypalmitate with a ΔG of −5.25 kcal/mol, but was not predicted to form a stable complex with palmitoyl-CoA (positive ΔG value). JmGELP-3 showed predicted to bind both substrates, with ΔG values of −6.25 kcal/mol for palmitoyl-CoA and −5.92 kcal/mol for 10,16-dihydroxypalmitate.

## 3. Discussion

The GELP gene family plays a central role in plant lipid metabolism and stress adaptation [8]. Its functional diversity provides a molecular basis for plants to coordinate growth, development, and environmental responses. However, most functional studies of *GELP* genes have focused on herbaceous model plants such as *Arabidopsis* [6], rice [3], watermelon [26], and tomato [13]. Information from woody perennials remains scarce, even though a number of studies have been published. In this study, we identified 61 *JmGELP* genes in *Juglans mandshurica*. Reported GELP family sizes vary considerably among woody perennials, including 87 in pecan [12], 83 in grape [27], 120 in macadamia [28], and 38 in apple [29]. This comparison suggests that GELP family size in woody dicots may vary with genome complexity and life history strategy and does not reflect a uniform expansion pattern.

The physicochemical properties of JmGELP proteins further support their putative roles as secreted lipolytic enzymes. The predominantly negative GRAVY values, together with the extracellular localization predicted for 93.44% of members, are consistent with the classical features of plant GELP enzymes, which are typically secreted to the apoplast to act upon extracellular lipid substrates [11]. The median aliphatic index of 85.5 suggests moderate thermostability, contributing to the environmental adaptability of *J. mandshurica* as a temperate tree species. Most JmGELPs are predicted to be stable proteins, with the exception of four members (JmGELP-26, -29, -51, and -59) that exhibit instability indices above the stability threshold (II > 40). The instability of these specific members may have functional significance, as plant GDSL esterases/lipases are known to participate in developmental processes and stress responses that often involve rapid protein turnover [8,19]. Furthermore, the broad pI range (4.23–9.37) reflects considerable charge heterogeneity among JmGELP members, which may contribute to functional diversification in substrate recognition and enzyme activity [2,10]. Collectively, these physicochemical features are consistent with the predicted characteristics of secreted lipolytic enzymes and suggest that JmGELP proteins may function in lipid metabolism and related physiological processes.

Phylogenetic analysis classified the 61 JmGELP members into seven subclades (Clust I–VII). This branching pattern is largely consistent with the subclade structure previously reported in other plant species [3,6,28], indicating that the evolutionary trajectory of this multigene family has been highly conserved across land plants, from mosses and conifers to angiosperms including rice and apple. The maintenance of this evolutionary architecture implies that subfamily divergence likely originated in the common ancestor of land plants and has been preserved under strong functional constraints. Notably, the distribution of JmGELP members among the seven subclades was uneven: Clust I (21 members) and Clust III (23 members) collectively accounted for more than 72% of all JmGELP members, whereas Clust IV–VII contained only 11 members in total. This uneven distribution suggests that certain subclades underwent lineage-specific expansion in *J. mandshurica*, potentially reflecting subfunctionalization or neofunctionalization driven by the species’ ecological adaptations [8,28]. Based on comparisons with functionally known homologs in *Arabidopsis*, we made initial functional predictions for *JmGELP* genes. Clade I members grouped with *AtGLIP2* [30], a GPAT-associated gene involved in glycerolipid synthesis, hinting at their potential roles in lipid metabolism and kernel oil accumulation. Given that Clade I is the largest subclade in terms of total GELP members (21 JmGELPs and 28 AtGELPs), this suggests that lipid metabolism-related functions may represent a major functional specialization within the JmGELP family. Clade II members clustered tightly with the defense-related gene *AtGLIP1* (*AT5G40990*) [31], suggesting that they may be involved in pathogen defense in *J. mandshurica*. Clade III, which contains the highest number of JmGELP members (23), may represent another functionally important group that has undergone preferential expansion in *J. mandshurica* [11]. These functional inferences are based solely on phylogenetic conservation and sequence homology. Further experimental validation is required to confirm these biological roles in the future.

Gene duplication drives the expansion and functional differentiation of gene families [32]. In *J. mandshurica*, the GELP family expanded mainly through whole-genome/segmental and dispersed duplication. This pattern is consistent with that observed in apple [29] and soybean [33], where WGD and segmental duplication dominate GELP family expansion. In contrast, rice [3] and *Arabidopsis* [6] show a predominance of tandem duplication. These differences may reflect divergent evolutionary strategies of the GELP family across plant lineages.

All JmGELP proteins contain the conserved GDSL motif and Ser-Asp-His catalytic triad, consistent with the structural basis for esterase/lipase activity. This feature has also been observed in pear and cucumber [4,9], indicating strong functional constraints on the conservation of core functions. Despite shared core features, the JmGELP family exhibits structural divergence. Variations in conserved motifs, exon–intron organization, and signal peptides contribute to this differentiation among members. Most members (57 of 61) were predicted to be secretory proteins, aligning with known GELP functions in cell wall modification and cuticle formation [9]. In *Arabidopsis* [6], pecan [12], and watermelon [26], GELP proteins are mainly targeted to the extracellular matrix. Four members in *J. mandshurica* were predicted to localize to the chloroplast, mitochondrion, or vacuole. Taken together, this subcellular heterogeneity suggests that the JmGELP family may coordinate lipid metabolic processes across multiple organelles, broadening their functional roles beyond the secretory pathway. JmGELP-51, which has an unusually long sequence and high instability index, may act as a transient regulatory factor, but this hypothesis requires testing.

Many *JmGELP* promoters contain Light-, hormone-, and stress-related cis-elements, suggesting that these family members respond to developmental and environmental signals. Transcriptome data showed that many *JmGELP* genes are differentially expressed across tissues and seed developmental stages. For example, genes highly expressed in early seed stages (e.g., *JmGELP-16*, *-22*, *-41*) may participate in embryogenesis, whereas genes upregulated in late stages (e.g., *JmGELP-13*, *-38*, *-48*) may be involved in seed filling and oil accumulation. Their downregulation at stage S4 coincides with seed maturation. Similar patterns have been observed in other woody oil plants. In pecan, some GELP genes are strongly induced by salt stress and peak during kernel maturation [12]. In macadamia, the large GELP family contains many ABA and MeJA response elements in its promoters [28]. Together, these observations suggest that oil-bearing woody perennials may also use GELP members for reproductive lipid deposition and stress-related barrier maintenance.

GELP family members are known to participate in cutin and suberin monomer biosynthesis and deposition [4,34]. Cutin and suberin are lipid-derived polymers that form physical barriers [35]. These barriers block pathogens and reduce water loss [4,9]. Our PPI analysis predicted that JmGELPs interact with lipid transfer proteins and peroxidases, and GO enrichment pointed to lipid catabolic process as the top term. Together, these results suggest that JmGELPs may be involved in lipid metabolism associated with barrier formation. To test this possibility, we then selected palmitoyl-CoA and 10,16-dihydroxypalmitate as candidate ligands, as both are intermediates in cutin and suberin pathways. Molecular docking provided additional computational evidence supporting these predicted interactions. The convergence of PPI and docking data suggests that JmGELPs might process barrier precursors. Nevertheless, docking alone is not proof; in vivo lipid composition analysis and direct enzyme assays will be required for validation.

Six *JmGELP* genes (*JmGELP-3*, *-4*, *-21*, *-25*, *-38*, *-41*) were selected for qRT-PCR validation. Based on their response patterns across four treatments (salt, PEG, SA, MeJA), three non-overlapping representative candidates were prioritized: *JmGELP-38* (strongest osmotic response, 8.0 × PEG), *JmGELP-41* (hormone-specific, 3.6 × SA, early seed development peak), and *JmGELP-3* (leaf-specific, mild but distinct salt/drought response, MeJA-repressed). Genes with redundant or weaker responses (*JmGELP-21*, *-25*, *-4*) were excluded. To further justify the selection of these three genes for subsequent bioinformatic analyses, we integrated phylogenetic relationships, cis-element profiles, expression patterns, and literature-derived functional evidence. These three candidates were chosen to represent the two major functional subclades identified in the phylogenetic analysis: JmGELP-38 and JmGELP-41 belong to Clust I (lipid metabolism-related), while JmGELP-3 belongs to Clust II (defense-related).

*JmGELP-3*, which falls within Clust II, was selected based on its evolutionary relationship with defense- and drought-responsive GDSL lipases. This clade contains the well-characterized defense regulators *AtGLIP1* (*AT5G40990.1*) and *AtGLIP2*, which mediate resistance against fungal and bacterial pathogens, respectively [30,31]. Importantly, the ortholog of *AtGLIP1* in pepper, *CaGLIP1*, has been functionally validated as a positive regulator of drought tolerance; its overexpression confers enhanced tolerance to drought stress [18]. *JmGELP-3* is mainly expressed in leaves and is barely detectable in fruit and bark. Its protein is predicted to bind both palmitoyl-CoA and 10,16-dihydroxypalmitate. Together with its mild stress induction, these features suggest that this gene may be involved in basal lipid turnover.

*JmGELP-38* and *JmGELP-41*, both belonging to Clust I, were selected based on multiple lines of evidence linking this subclade to lipid metabolism and stress adaptation. *JmGELP-38* is closely related to *AT5G14450.1*, which is the ortholog of the soybean GDSL lipase *GmGELP28*; overexpression of *GmGELP28* has been shown to enhance drought tolerance in soybean [33]. *JmGELP-38* peaks in expression during late seed development. Its promoter is enriched in ERF and NAC binding motifs, and the gene is strongly induced by drought stress. Similar drought-induced expression has been reported for *GELP* genes in other woody plants. For instance, overexpression of *CpGLIP1* from *Chimonanthus praecox* enhanced drought and cold tolerance in poplar and *Arabidopsis* [36]. However, most of those studies remain at the expression level, and the underlying mechanisms are still unclear.

*JmGELP-41* is closely related to *AT5G03610.1* (*GGL25*), which is downregulated under waterlogging stress in pennycress roots, implicating its involvement in osmotic stress response and modification of extracellular structures such as suberin [37]. Additionally, *AT1G54790.1* (*AtGELP26*), which clusters with *JmGELP-38* and *JmGELP-41* in the same subclade (Clust I), has been functionally characterized as a negative regulator of seed fatty acid accumulation and composition through the DELLA-mediated gibberellin signaling pathway [38]. *JmGELP-41* shows an expression peak at the early stage of seed development, followed by a rapid decline. This timing overlaps with the key window for epidermal barrier establishment. A similar early-stage expression pattern was reported for *BnSCE3*/*BnLIP2* in *Brassica napus* [39], whose overexpression promotes germination and seedling growth; however, this role lacks support from loss-of-function mutants. The same pattern may apply to *JmGELP-41* in early seed development. In addition, *JmGELP-41* is induced by SA and MeJA. Taken together, these features suggest that *JmGELP-41* may participate in both early seed development and defense-related lipid metabolism. However, this remains a hypothesis and requires experimental validation.

This study characterizes the GELP family in *J. mandshurica* and suggests functional differentiation among members. However, most conclusions come from bioinformatics. Transcriptome and qRT-PCR data provide correlative evidence only. We acknowledge that the qRT-PCR analysis was performed at a single time point (24 h), which may not capture the full temporal dynamics of gene expression. Stable transformation is not yet available for this species. Therefore, the proposed roles of *JmGELP-3*, *-38*, and *-41* remain speculative. Future work should express *JmGELP* genes in model plants (e.g., *Arabidopsis* or poplar) and test their functions under stress, and incorporate multi-time-point analyses (e.g., 0, 6, 12, 24, 48 h) to fully characterize their stress-responsive kinetics.

## 4. Materials and Methods

### 4.1. Plant Material

Leaf samples were collected from *Juglans mandshurica* trees growing in the resource garden of Xi’an Botanical Garden of Shaanxi Province (109.03° E, 34.21° N, Xi’an, China) on 20 August 2025. Voucher specimens were identified by Professor Huijuan Zhou. To eliminate the effects of circadian rhythm, all samples were obtained at the same time of day. Three biological replicates were collected for each treatment.

### 4.2. Identification of GELP Gene Members in J. mandshurica

To identify GELP members in *J. mandshurica*, we first retrieved all GELP protein sequences of *A. thaliana* from the TAIR database (http://www.arabidopsis.org/). These sequences were used as queries for a genome-wide BLASTP search (NCBI BLAST+ v.2.16.0) against the *J. mandshurica* genome with an E-values threshold of 1 × 10^−5^ [25]. Subsequently, the protein structural domains were analyzed using the NCBI Conserved Domain Database (https://www.ncbi.nlm.nih.gov/cdd/ (accessed on 3 September 2025)), Pfam database in InterPro (https://www.ebi.ac.uk/interpro/search/sequence/ (accessed on 4 September 2025)), and SMART database (http://smart.embl.de/ (accessed on 4 September 2025)), respectively. The candidate genes that contain the conserved domain SGNH_plant_lipase_like (PF00657) and the GDSL catalytic triplet structure were preliminarily considered to be members of the JmGELP gene family. The filtered protein sequences were then analyzed using MEME (https://meme-suite.org/meme/tools/meme (accessed on 6 September 2025)) for motif detection, and those containing the GDSL motif were designated as final JmGELP members.

### 4.3. Prediction of the Physicochemical Properties and Subcellular Localization of the JmGELP Gene Family

Physicochemical properties of all identified JmGELP proteins in *J. mandshurica* were predicted using the Protein Parameter Calc plugin in TBtools v.2.467 [40]. Subcellular localization was predicted using the plant-mPLOC (http://www.csbio.sjtu.edu.cn/bioinf/plant-multi/ (accessed on 9 September 2025)) and TargetP-2.0 (https://services.healthtech.dtu.dk/services/TargetP-2.0/ (accessed on 9 September 2025)).

### 4.4. Phylogenetic Analysis, Conserved Domains, Motifs and Gene Structures of the JmGELP Gene Family

To investigate the evolutionary relationships and functional divergence of the GELP gene family in *J. mandshurica*, we constructed a phylogenetic tree using the 61 JmGELP proteins together with 100 GELP homologs from *A. thaliana*. The inclusion of *A. thaliana* homologs allowed us to infer the evolutionary relationships between JmGELP and AtGELP members and to make initial functional predictions for uncharacterized *JmGELP* genes based on the known functions of their closely related *AtGELP* counterparts. A maximum likelihood (ML) phylogenetic tree based on the protein sequences of GELP families from *J. mandshurica* and *A. thaliana* was constructed using IQ-tree v.2 software (Bootstrap: 1000; Best BIC score model: JTT + R10) [41]. The phylogenetic tree was visualized using the iTOL v.5 online website (https://itol.embl.de/login.cgi/ (accessed on 10 September 2025)) [42]. Conserved domains and motifs of GELP were analyzed and visualized using NCBI CD-search (https://www.ncbi.nlm.nih.gov/cdd/ (accessed on 11 September 2025)), MEME (https://meme-suite.org/meme/tools/meme (accessed on 11 September 2025)), and TBtools v.2.467 [40]. TBtools v.2.467 was used to integrate the evolutionary tree of JmGELPs with conserved domains and motifs [40]. We submitted the identified JmGELP family members to the online website of Gene Structure Display Server 2.0 (http://gsds.gao-lab.org/ (accessed on 11 September 2025)) for gene structure analysis. Gene locations were visualized using the Gene Location Visualizer plugin in TBtools v.2.467 [40].

### 4.5. Chromosomal Distribution and Synteny Analysis of JmGELP Genes

*JmGELP* genes were mapped to the chromosome based on the *J. mandshurica* genomic data. Chromosomal distribution diagrams were generated using MG2C online software v2.0 (http://mg2c.iask.in/mg2c_v2.0/ (accessed on 15 September 2025)). Duplication events were analyzed using Multiple Collinearity Scan toolkit X v1.0.0 (MCScanX) with the default parameters [43]. Synteny analysis of GELP genes was performed between *J. mandshurica* and *A. thaliana*, and between *J. mandshurica* and *J. regia*, with TBtools v.2.467 used for visualization of syntenic relationships [40]. In addition, TBtools v.2.467 was used to compute the non-synonymous (Ka) and synonymous (Ks) substitution rates for duplicated gene pairs [40].

### 4.6. Cis-Acting Element Prediction and Statistical Analysis

Cis-acting element prediction was performed using 2000 bp sequences upstream of the identified GELP genes coding sequence (CDS) at PlantCARE (http://bioinformatics.psb.ugent.be/webtools/plantcare/html/ (accessed on 16 September 2025)). The results were visualized with an R script (R v.4.3.2) after statistical screening.

### 4.7. Expression Profiling of JmGELP Genes Across Tissues and During Fruit Development

To investigate the expression patterns of the JmGELP gene family, we analyzed publicly available RNA-seq data from *J. mandshurica* [25,44]. The datasets comprised two BioProjects: PRJNA816294 (tissues: bark, green fruit pericarp, and leaf) and PRJNA733587 (embryo stages S1–S4). All libraries were sequenced on the Illumina platform to a depth of >20 million paired-end reads per sample. Three biological replicates were used for leaf, bark, fruit pericarp, and embryo stages (S1–S4) (see Table S6 for details). Downloaded SRA files were converted to FASTQ format. Raw reads were quality-trimmed using Trimmomatic with parameters “LEADING:3 TRAILING:3 SLIDINGWINDOW:4:15 MINLEN:36”. The cleaned reads were then aligned to the *J. mandshurica* reference genome using HISAT2 v.2.2.1 to generate BAM files [45]. Transcript abundance was quantified at the gene level using featureCounts v2.1.1 via an R script (run-featurecounts.R) to obtain a raw count matrix. The RNA-seq libraries were treated as non-strand-specific, and featureCounts v2.1.1 was run with the default -s 0 parameter. Differentially expressed genes (DEGs) were identified using a Perl script (run_DE_analysis.pl) with Perl v5.32.1, implementing the DESeq2 v1.42.1 method. The significance threshold was set to false discovery rate (FDR) < 0.05 and |log_2_FoldChange| > 1. Heatmaps depicting the expression of JmGELP DEGs were generated using TBtools v.2.467 [40].

### 4.8. Validation by Quantitative Real-Time PCR (qRT-PCR)

To explore the potential roles of *JmGELP* genes in response to abiotic stresses and hormone signaling, qRT-PCR was performed on six selected leaf-expressed genes. For treatments, entire leaves were immersed in solutions containing 200 mM NaCl (salt stress), 20% (*w*/*v*) PEG-6000 (drought-mimetic stress), 100 μM salicylic acid (SA), and 100 μM methyl jasmonate (MeJA). Treatments were carried out in Petri dishes with gentle shaking (80 rpm) at 25 °C under a 16 h light/8 h dark photoperiod for 24 h. This single time point was selected to compare the relative responsiveness of candidate genes across treatments under a unified condition. Control leaves were immersed in distilled water (with 0.01% ethanol for hormone controls) under identical conditions. After 24 h, all samples (including controls) were collected, immediately frozen in liquid nitrogen, and stored at −80 °C for total RNA extraction. RNA integrity, purity, and concentration were assessed by agarose gel electrophoresis and a NanoDrop™ spectrophotometer (Thermo Fisher Scientific, Waltham, MA, USA). Based on transcriptome data, six highly expressed *JmGELP* genes in leaves (*JmGELP-3*, *-4*, *-21*, *-25*, *-38*, and *-41*) were selected for expression analysis. Their primer sequences are listed in Supplementary Table S8. The β-Actin gene was used as an internal reference. Gene-specific primers were designed using the Primer3Plus online tool (https://www.primer3plus.com*/* (accessed on 15 November 2025)). qRT-PCR reactions were conducted on a Bio-Rad CFX96 Real-Time PCR System (Bio-Rad, Hercules, CA, USA) with three technical replicates per reaction. Relative gene expression levels were calculated using the 2^−ΔΔCT^ method [46].

### 4.9. Protein–Protein Interaction Network Construction and Analysis

To systematically explore potential functional modules within the JmGELP gene family, we predicted a protein–protein interaction (PPI) network. These homologous *A. thaliana* GELP genes were used as proxies to search the STRING database (https://string-db.org/ (accessed on 25 December 2025)) and retrieve high-confidence interaction data with a combined score threshold ≥0.700. Functional enrichment analysis of key network modules was performed to infer their potential biological roles (https://string-db.org/ (accessed on 25 December 2025)).

### 4.10. Prediction of Transcription Factor Binding Sites and microRNA Targets

Promoter sequences (2000 bp upstream of the transcription start site) were extracted for each candidate *JmGELP* gene and submitted to the PlantRegMap online tool (http://plantregmap.gao-lab.org/ (accessed on 27 December 2025)) for genome-wide prediction of cis-regulatory elements and their cognate transcription factors (TFs). MicroRNA targeting prediction was performed for the candidate *JmGELP* genes using default parameters from the psRNATarget online website (https://www.zhaolab.org/psRNATarget/ (accessed on 27 December 2025)). The network was visualized using Cytoscape v.3.10.0 with default parameters [47].

### 4.11. Molecular Docking Analysis of JmGELP Protein Models

The three-dimensional structures of candidate JmGELP proteins were obtained from the AlphaFold predicted models via the UniProt online platform (https://www.uniprot.org/ (accessed on 7 January 2026)). No experimental structures were available in the Protein Data Bank (PDB) (https://www.rcsb.org/ (accessed on 7 January 2026)). The predicted protein structures were preprocessed using PDBFixer tool (https://github.com/openmm/pdbfixer/ (accessed on 10 January 2026)) to add hydrogen atoms and optimize protonation states for biological relevance. The predicted active site cavity was defined based on the conserved catalytic triad (Ser, Asp, His) characteristic of the GELP gene family. A grid box of 20 × 20 × 20 Å was centered at the geometric center of the catalytic triad residues to fully encompass the active pocket region [48]. The 2D structures of the two ligand molecules were obtained from the PubChem database (https://pubchem.ncbi.nlm.nih.gov/ (accessed on 10 January 2026)). The PubChem CIDs were 644109 for palmitoyl-CoA and 25201518 for 10,16-dihydroxypalmitate. Ligands were converted to the PDBQT format and energy-minimized using OpenBabel v.3.1.1 prior to docking. Molecular docking calculations were carried out using AutoDock Vina v.1.2.3, with the catalytic triad defining the active site and all other parameters left at their default values [48]. For each docking run, the conformation with the lowest binding energy was selected as the optimal result. Three-dimensional overviews and detailed interaction diagrams of the docking models were generated using PyMOL v.3.1 (https://www.pymol.org/ (accessed on 20 January 2026)), while 2D interaction diagrams were produced using LigPlot+ v.2.2.5 [49].

## 5. Conclusions

We identified and characterized the GELP gene family in *Juglans mandshurica*. A total of 61 JmGELP members were classified into seven major clades. Variations in gene structure, promoter cis-elements, and expression patterns, combined with PPI network evidence, suggest functional divergence among family members, particularly in lipid metabolism and stress adaptation. Three candidate genes (*JmGELP-3*, *-38*, and *-41*) exhibited distinct spatiotemporal expression profiles and different stress response behaviors. Compared with *JmGELP-3*, both *JmGELP-38* and *JmGELP-41* harbored more transcription factor and miRNA binding sites, and, and were predicted to possess distinct substrate binding preferences based on in silico docking analysis. These differences suggest that the three genes may have distinct regulatory mechanisms and functional orientations. Collectively, our results provide a basic genomic and expression resource for the JmGELP family in *J. mandshurica*. The three candidate genes may represent valuable targets for future functional studies on lipid metabolism and stress responses in *J. mandshurica*, although their proposed roles remain hypothetical and require experimental validation through methods such as heterologous expression or gene silencing.

## Figures and Tables

**Table 1:** Characterization of the GELP gene family members in *Juglans mandshurica*.

Gene	No. of Amino Acid (AA)	Mol. Wt (Da)	Iso-Electric Point (PI)	Instability Index (II)	Aliphatic Index	Grand Average of Hydropathicity (GRAVY)	Subcellular Location
*JmGELP-1*	359	38,530.02	8.79	23.69	92.98	−0.004	Extracell
*JmGELP-2*	364	39,796.39	9.17	28.06	77.2	−0.279	Extracell
*JmGELP-3*	398	44,068.72	8.18	28.21	86.76	−0.042	Extracell
*JmGELP-4*	373	41,021.8	6.6	24.51	88.42	0.01	Extracell
*JmGELP-5*	380	42,944.23	9.37	32.42	83.63	−0.23	Extracell
*JmGELP-6*	427	46,488.5	5.42	31.54	98.2	0.253	Extracell
*JmGELP-7*	392	42,741.89	6.58	30.53	84.36	0.074	Extracell
*JmGELP-8*	376	41,083.24	8.57	25	92.66	−0.056	Extracell
*JmGELP-9*	350	39,462.94	6.09	36.49	82.23	−0.158	Extracell
*JmGELP-10*	380	41,943.03	5.8	37.85	91.92	0.012	Extracell
*JmGELP-11*	394	43,453.08	5.25	36.43	81.22	−0.181	Extracell
*JmGELP-12*	345	37,911.07	5.24	35.75	88.52	0.015	Extracell
*JmGELP-13*	356	38,393.46	6.31	33.18	91.07	−0.067	Extracell
*JmGELP-14*	386	42,526.99	7.15	36.09	79.92	−0.166	Extracell
*JmGELP-15*	382	42,666	6.31	35.14	76.41	−0.291	Extracell
*JmGELP-16*	383	42,593.29	6.79	36.33	80.97	−0.242	Extracell
*JmGELP-17*	363	40,590.76	8.97	35.34	75.29	−0.282	Extracell
*JmGELP-18*	375	40,942.67	7.54	24.95	87.41	0.013	Extracell
*JmGELP-19*	390	43,038.85	8.76	38.03	81.56	−0.203	Extracell
*JmGELP-20*	400	44,257.44	6.45	31.08	85.85	−0.071	Mitochondrion
*JmGELP-21*	386	42,539.56	7.99	36.74	80.36	0.001	Extracell
*JmGELP-22*	386	42,839.76	8.7	32.57	75.28	−0.155	Extracell
*JmGELP-23*	374	41,395.85	4.9	32.95	83.48	−0.179	Extracell
*JmGELP-24*	375	41,785.58	8.77	32.69	82.13	−0.105	Extracell
*JmGELP-25*	367	40,400.18	8.92	30.96	87.11	−0.104	Extracell
*JmGELP-26*	394	43,696.18	8.38	33.74	89.11	0.012	Extracell
*JmGELP-27*	412	45,016.28	4.95	48.27	85.46	−0.052	Chloroplast
*JmGELP-28*	468	51,774.18	6.25	28.01	84.04	0.024	Extracell
*JmGELP-29*	378	42,093.44	6.13	40.9	91.9	0.075	Extracell
*JmGELP-30*	362	39,823.27	5.15	34.68	96.74	0.04	Extracell
*JmGELP-31*	389	42,782.45	5.6	32.97	79.56	−0.062	Extracell
*JmGELP-32*	368	41,056.33	8.93	32.54	95.35	0.017	Extracell
*JmGELP-33*	374	40,613.74	5.04	30.95	98.56	0.11	Extracell
*JmGELP-34*	370	40,576.06	4.92	32.05	82.84	−0.012	Extracell
*JmGELP-35*	362	40,400.45	8.25	38.16	87.6	0.067	Extracell
*JmGELP-36*	361	40,133.85	5.35	24.3	85.12	−0.012	Extracell
*JmGELP-37*	384	41,602.79	9.3	24.55	89.87	−0.031	Extracell
*JmGELP-38*	380	42,464.17	8.28	38.07	78.5	−0.273	Extracell
*JmGELP-39*	369	40,491.08	8.72	29.95	79.67	−0.027	Extracell
*JmGELP-40*	367	40,666.33	8.7	39.47	90.82	0.063	Extracell
*JmGELP-41*	372	41,838.5	9.18	35.65	74.65	−0.333	Extracell
*JmGELP-42*	349	38,458.01	8.8	26.08	86.25	−0.013	Extracell
*JmGELP-43*	338	36,620.6	6.93	29.04	94.97	0.093	Extracell
*JmGELP-44*	374	41,270.24	4.8	32.86	80.88	−0.244	Extracell
*JmGELP-45*	356	39,173.21	4.3	32.28	82.98	0.007	Extracell
*JmGELP-46*	356	39,099.08	4.23	29.69	84.07	0.025	Extracell
*JmGELP-47*	383	42,470.13	8.02	30.84	78.69	−0.163	Extracell
*JmGELP-48*	359	38,926.75	9.37	29.17	84.85	−0.055	Extracell
*JmGELP-49*	385	43,171.49	9.34	32.93	79.3	−0.185	Extracell
*JmGELP-50*	390	42,967.59	7.06	37.89	81.08	−0.217	Mitochondrion
*JmGELP-51*	551	58,741.29	6.92	67.53	61.67	−0.28	Extracell
*JmGELP-52*	368	40,153.69	8.07	31.53	85.73	−0.033	Extracell
*JmGELP-53*	369	40,275.81	8.07	30.28	87.62	−0.022	Extracell
*JmGELP-54*	362	40,297.06	6.31	33.81	87.82	−0.11	Extracell
*JmGELP-55*	354	38,979.55	8.41	38.25	92.91	0.207	Extracell
*JmGELP-56*	376	41,990.59	6.44	31.22	93.38	0.176	Extracell
*JmGELP-57*	357	39,305.15	5.75	36.39	92.04	0.021	Extracell
*JmGELP-58*	372	40,934.93	9.07	33.72	95.46	−0.062	Extracell
*JmGELP-59*	443	49,238.11	5.16	45.85	89.8	0.093	Vacuole
*JmGELP-60*	387	42,407.8	8.53	32.35	72.09	−0.122	Extracell
*JmGELP-61*	371	41,637.33	8.42	35.62	99.65	0.018	Extracell

## Data Availability

The raw genome sequence data and the RNA-seq datasets analyzed in this study are publicly available. The genome data can be accessed via the Genome Warehouse (NGDC) under accession number PRJCA006358 (https://ngdc.cncb.ac.cn/bioproject/browse/PRJCA006358, accessed on 13 April 2025). The RNA-seq datasets are deposited in the NCBI SRA database under accession numbers PRJNA816294 and PRJNA733587. All processed data generated in this study are provided in the Supplementary Materials. Further inquiries can be directed to the corresponding author.

## Supplementary Materials

The following supporting information can be downloaded at https://www.mdpi.com/article/10.3390/ijms27156557/s1.
